# The Prognostic Significance of Proteinuria Severity in Pregnancy: A Retrospective Cohort Study of Maternal and Neonatal Outcomes

**DOI:** 10.3390/jcm15010345

**Published:** 2026-01-02

**Authors:** Barış Boza, Fırat Ersan, Verda Alpay, Hakan Erenel

**Affiliations:** Department of Obstetrics and Gynecology, Division of Perinatology, Başakşehir Çam and Sakura City Hospital, Istanbul 34480, Turkey; phratersan@yahoo.com (F.E.); verda_alpay@yahoo.com (V.A.); hakanerenel@yahoo.com (H.E.)

**Keywords:** proteinuria, pregnancy, adverse maternal outcomes, preeclampsia

## Abstract

**Objective**: To investigate the impact of proteinuria severity on obstetric and neonatal outcomes and to assess the predictive value of 24 h urinary protein excretion, both alone and within a multivariable model, for adverse pregnancy outcomes. **Methods**: This retrospective cohort study included 203 pregnant women with proteinuria who were classified into mild (≥0.3 g/day and <3.0 g/day, *n* = 50), severe (≥3.0 g/day and <5.0 g/day, *n* = 67), and massive (≥5.0 g/day; *n* = 86) groups based on 24 h urine protein levels. Maternal and neonatal outcomes were compared between these groups. Correlation analysis, receiver operating characteristic (ROC) curve analysis, and multivariable logistic regression were used to evaluate the predictive value of proteinuria for obstetric complications and identification of increased risk of early delivery. The AUC values of the proteinuria-only model and the multivariable model were compared using the DeLong test, as both models were derived from the same dataset and therefore represented correlated ROC curves. **Results**: The incidence of obstetric complications was significantly higher in the severe (68.7%) and massive (81.4%) proteinuria groups compared with the mild group (32.0%; *p* < 0.001). Increasing proteinuria severity was associated with earlier gestational age at delivery, lower birth weight, and higher rates of fetal growth restriction (all *p* < 0.001). The 24 h proteinuria level demonstrated moderate predictive ability for obstetric complications (AUC 0.73; 95% CI 0.66–0.80). A multivariable model including nephrotic-range proteinuria (≥3 g/day) and gestational age at diagnosis showed improved discriminatory performance compared with proteinuria alone (AUC 0.81; 95% CI 0.75–0.88). The model based on continuous 24 h proteinuria yielded an AUC of 0.73 (95% CI, 0.66–0.80) for identifying pregnancies at increased risk of obstetric complications. The multivariable model showed a numerically higher AUC of 0.81 (95% CI, 0.73–0.86); however, the difference between the two AUCs was not statistically significant according to the DeLong test (z = 0.82, *p* = 0.41). **Conclusions**: The severity of maternal proteinuria is associated with a higher likelihood of adverse maternal and neonatal outcomes, and higher proteinuria levels appear to show a graded association with increasing risk. A multivariable model integrating proteinuria with key clinical parameters demonstrated moderate discriminatory ability for obstetric complications, may support a more holistic approach to risk stratification in clinical practice.

## 1. Introduction

Proteinuria is a common and clinically important finding in pregnancy. It may reflect a wide range of conditions, from mild and temporary changes to severe and life-threatening maternal diseases [1,2]. Normal physiological changes during pregnancy, such as an increase in glomerular filtration rate of up to 50%, cause a natural rise in urinary protein excretion. However, proteinuria greater than 300 mg in a 24 h urine collection is considered abnormal and is an important criterion in the diagnosis of hypertensive disorders of pregnancy, especially preeclampsia [3,4]. Although the 24 h urine collection is still the gold standard for measuring proteinuria, simpler methods such as the spot urine protein-to-creatinine ratio (PCR) are often used in clinical practice, with a cut-off value of ≥0.3 to indicate significant proteinuria [5,6].

The clinical context of proteinuria varies widely. Although it is most commonly associated with preeclampsia—a multisystem disorder and a leading cause of maternal and perinatal morbidity and mortality—other conditions should also be considered in the differential diagnosis. These include gestational proteinuria, which is generally regarded as a benign condition, and worsening of pre-existing kidney disease or chronic hypertension during pregnancy [7,8]. In addition, although less common, non-obstetric causes may present with significant proteinuria, including rare but serious conditions such as de novo nephrotic syndrome and atypical hemolytic uremic syndrome (a-HUS), which occur in approximately 0.012–0.025% of pregnancies [9,10]. This wide range of potential causes highlights the diagnostic challenge and the critical need for accurate risk stratification.

A major and ongoing debate in perinatal medicine concerns the independent prognostic value of proteinuria severity. Although its role as a diagnostic marker for preeclampsia is well established, the clinical significance of proteinuria magnitude beyond the initial diagnostic threshold remains the subject of considerable research and discussion [11]. Numerous studies have demonstrated a strong, dose-dependent association between increasing levels of proteinuria—commonly classified as mild (<3 g/day), severe (3–4.9 g/day), and massive or nephrotic-range (≥5 g/day)—and a higher incidence of adverse maternal and neonatal outcomes [12,13]. These adverse outcomes include increased rates of preterm birth, fetal growth restriction (FGR), low birth weight, and other obstetric complications [14,15]. For example, some studies suggest that nephrotic-range proteinuria is a significant predictor of composite adverse perinatal outcomes, even when its association with adverse maternal outcomes is less consistent [16].

On the other hand, some clinical guidelines and studies question how useful the amount of proteinuria is for assessing the severity of preeclampsia or guiding patient management. They suggest that proteinuria is a weaker predictor of complications compared with other clinical findings, such as maternal blood pressure or signs of liver and blood-related problems [1,11]. According to this view, once preeclampsia is diagnosed, the level of protein excretion adds little to immediate clinical decision-making. As a result, clinical practice has varied, with some clinicians continuing to monitor proteinuria closely, while others focus more on a wider range of clinical and laboratory findings to evaluate disease progression.

This debate highlights several important gaps in the current literature. First, while many studies focus on proteinuria related to preeclampsia, there is limited information on outcomes associated with severe proteinuria during pregnancy due to non-obstetric causes. Second, the ability of 24 h urine protein measurement alone to predict outcomes has not been clearly established across the full range of proteinuric disorders in pregnancy, especially when compared with multivariable models that include other easily available clinical parameters. Finally, identifying clinically meaningful thresholds of proteinuria severity—particularly at nephrotic-range levels—that could reliably improve risk assessment and prenatal management remains an important goal.

For this reason, this retrospective study was designed to examine the effect of proteinuria severity on obstetric and neonatal outcomes in pregnant women followed at a tertiary perinatology clinic. We compared outcomes among patients with mild, severe, and massive proteinuria. The aims of the study were to: (1) examine the distribution of underlying causes and the frequency of obstetric complications and the need for antihypertensive treatment; (2) evaluate how proteinuria severity affects important neonatal outcomes, such as gestational age at delivery, birth weight, and the rate of fetal growth restriction (FGR); and (3) assess the ability of 24 h urine protein levels to define discriminatory ability for obstetric complications and preterm birth, both alone and when combined with other clinical factors, using correlation analysis and ROC curve analysis.

## 2. Materials and Methods

This retrospective cohort study was conducted at a single tertiary perinatology clinic over a five-year period, from January 2020 to January 2025. Medical records of all pregnant patients who were diagnosed with and treated for proteinuria were reviewed. The study was conducted in accordance with the Declaration of Helsinki, and the protocol was approved by the Ethics Committee of Başakşehir Çam and Sakura City Hospital Scientific Research Ethics Committee No. 1 IRB number: 2025/302 on 8 October 2025. The research was conducted retrospectively using anonymized patient data. In our institution, patients routinely provide written consent at the time of hospital admission allowing the use of anonymized clinical data for research purposes; therefore, no additional study-specific consent was required.

**Figure 1:**
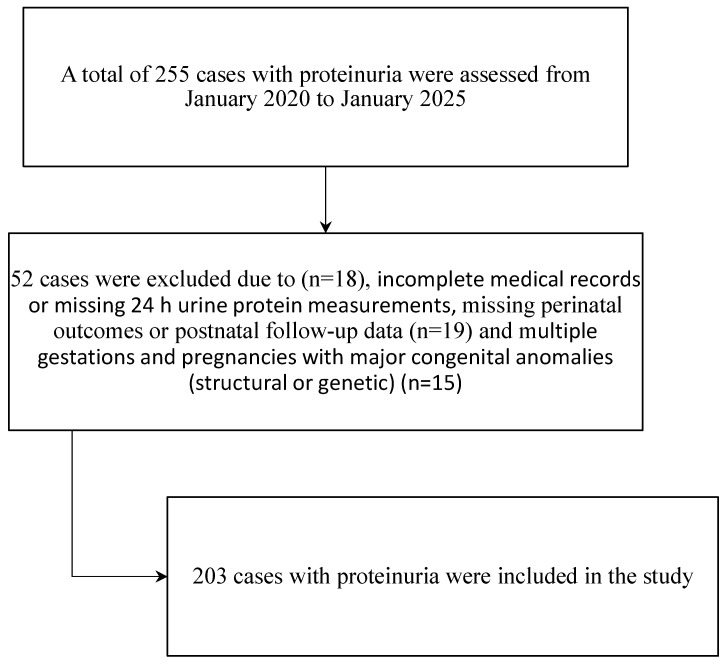
Flowchart of case screening, exclusion criteria, and final cohort formation.

The inclusion criteria covered all pregnant women with significant proteinuria, defined as a 24 h urine protein excretion of ≥300 mg, who subsequently delivered at our center. Patients with incomplete medical records or missing 24 h urine protein measurements were excluded from the analysis. Multiple gestations and pregnancies with major congenital anomalies (structural or genetic) were excluded from the study, as these conditions may independently increase the risk of fetal growth restriction and preterm birth. In total, 203 patients met the inclusion criteria and were included in the study. Because this study was conducted at a tertiary referral perinatology center, we acknowledge the potential for referral bias, whereby pregnancies complicated by more severe proteinuria or associated maternal–fetal conditions are more likely to be referred and managed at our institution. Conversely, women with mild proteinuria and an uncomplicated clinical course may deliver at secondary or primary care centers and therefore not be captured in our cohort. To enhance transparency and allow readers to assess the potential impact of this selection process, a detailed flow diagram illustrating patient screening, exclusions, and final inclusion is provided (Figure 1). Importantly, all eligible women with documented proteinuria who delivered at our center during the study period were consecutively included, minimizing selection bias within the defined source population.

Patients diagnosed with proteinuria were divided into three groups based on the severity of 24 h urine protein excretion, in line with commonly used clinical classifications [12,14]. For all analyses, the 24 h urine protein value used was the first complete 24 h urine collection obtained at the time of initial diagnosis of proteinuria and prior to delivery. In patients with hypertensive disorders of pregnancy, this measurement corresponded to the diagnostic evaluation before any decision regarding delivery timing was made. Peak or subsequent proteinuria measurements obtained later in the disease course were not used in the analyses. The groups were defined as follows:

Mild Proteinuria: 24 h urine protein excretion ≥ 0.3 g/day and <3.0 g/day (*n* = 50).

Severe Proteinuria: 24 h urine protein excretion ≥ 3.0 g/day and <5.0 g/day (*n* = 67).

Massive Proteinuria: 24 h urine protein excretion ≥ 5.0 g/day (*n* = 86).

Comprehensive data for each patient were obtained from electronic medical records. The collected variables included maternal demographic characteristics such as age, gravidity, parity, and body mass index (BMI), as well as mean arterial pressure (MAP) and key clinical parameters, including gestational age at diagnosis and at delivery.

The underlying cause of proteinuria was determined based on established diagnostic criteria through a careful review of clinical notes, laboratory findings, and discharge summaries. Diagnoses were categorized as preeclampsia, preeclampsia superimposed on chronic hypertension, gestational proteinuria, nephrotic syndrome, or hemolytic uremic syndrome (HUS). The etiological classification of proteinuria was based on established clinical and laboratory diagnostic criteria. Preeclampsia was defined according to the American College of Obstetricians and Gynecologists (ACOG) guidelines as new-onset hypertension after 20 weeks of gestation (systolic blood pressure ≥ 140 mmHg and/or diastolic blood pressure ≥ 90 mmHg on at least two occasions) accompanied by proteinuria (≥0.3 g/day) or, in the absence of proteinuria, evidence of end-organ dysfunction. Superimposed preeclampsia was diagnosed in women with pre-existing chronic hypertension who developed new-onset proteinuria or a sudden worsening of hypertension and/or systemic features of preeclampsia after 20 weeks of gestation.

Gestational proteinuria was defined as isolated proteinuria (≥0.3 g/day) arising after 20 weeks of gestation in the absence of hypertension or other features of preeclampsia, with resolution postpartum. Nephrotic syndrome was diagnosed based on nephrotic-range proteinuria (≥3–3.5 g/day) accompanied by hypoalbuminemia and/or edema, following evaluation by nephrology specialists and exclusion of preeclampsia-related renal involvement. Hemolytic uremic syndrome (HUS) was diagnosed based on the presence of microangiopathic hemolytic anemia, thrombocytopenia, and acute kidney injury, in accordance with established clinical criteria.

Patients with known chronic kidney disease prior to pregnancy were excluded based on medical history, baseline renal function, and pre-pregnancy records when available.

Composite obstetric complications (primary maternal outcome) were defined as the occurrence of any of the following prespecified events during the index pregnancy:

(1) Fetal distress requiring urgent delivery was operationalized according to standardized documentation criteria at our center, and was recorded only when expedited delivery was undertaken due to non-reassuring fetal heart rate patterns (e.g., recurrent late decelerations, persistent bradycardia, minimal/absent variability) and/or abnormal biophysical profile findings (BPP ≤ 4/10), as explicitly stated in the medical chart as the formal indication for delivery;
(2) Placental abruption, diagnosed clinically or confirmed intraoperatively;
(3) New onset of severe features of preeclampsia according to ACOG criteria (including severe hypertension, thrombocytopenia, hepatic or renal dysfunction, pulmonary edema, or neurological symptoms);
(4) HELLP syndrome;
(5) Intrauterine fetal death at ≥20 weeks of gestation, confirmed clinically or by ultrasound.

Only the first qualifying event per pregnancy was counted in the composite outcome. In addition, the need to start or increase antihypertensive treatment was also recorded.

Neonatal outcomes included gestational age at delivery, birth weight, APGAR scores at 1 and 5 min, and the presence of fetal growth restriction (FGR). FGR was defined according to the Delphi/ISUOG consensus criteria, using a combination of fetal size and Doppler parameters. Accordingly, FGR was diagnosed when EFW or AC < 3rd percentile, or when EFW/AC < 10th percentile accompanied by abnormal Doppler findings (elevated umbilical artery PI, absent/reversed end-diastolic flow, or low cerebroplacental ratio/abnormal MCA Doppler), consistent with gestational-age–specific criteria proposed by the consensus panel. Prematurity was classified by gestational age as extremely preterm (<28 weeks), very preterm (28 to <32 weeks), moderate preterm (32 to <34 weeks), late preterm (34 to <37 weeks), or term (≥37 weeks) [17].

### Statistical Analysis

Statistical analyses were performed using IBM SPSS Version 24.0 (Chicago, IL, USA). For selection of the appropriate statistical tests, the normality of continuous variables was assessed using histogram plots, evaluation of kurtosis and skewness values and performing the Kolmogorov–Smirnov and Shapiro–Wilk tests. Continuous variables that were normally distributed were presented as mean and ±standard deviation (SD) and for comparisons across the three groups (mild, severe, and massive proteinuria groups), a one-way analysis of variance (ANOVA) was used, followed by Tukey’s or Games–Howell post hoc tests as appropriate based on the homogeneity of variances assessed by Leven’s test. Variables that were not normally distributed are expressed as median (minimum-maximum) and were compared using Kruskal–Wallis H test and post hoc pairwise comparison was performed using Mann–Whitney U test. For the comparison of categorical variables, the Chi-Square and Fisher’s exact tests were performed. For group-to-group comparison, pairwise Chi-Square tests with Bonferroni correction were applied to identify which group differed significantly. A *p*-value of <0.05 was considered statistically significant and according to Bonferroni correction a *p*-value of <0.016 was considered statistically significant. Adjusted residuals were examined to determine which variables reached statistical significance in each group.

To evaluate correlations between the proteinuria levels and perinatal outcomes, Pearson’s and Spearman’s correlation analysis was performed.

To assess the robustness of the observed associations and to address potential confounding by etiologic heterogeneity, both sensitivity and stratified analyses were performed. In sensitivity analyses, the primary analyses were repeated after excluding pregnancies with non-obstetric renal causes of proteinuria, including nephrotic syndrome and hemolytic uremic syndrome, thereby restricting the cohort to obstetric causes of proteinuria (preeclampsia and superimposed preeclampsia). In addition, a separate sensitivity analysis was conducted in a preeclampsia-only cohort.

For stratified analyses, the study population was divided a priori into two major etiologic categories: hypertensive disorders of pregnancy (preeclampsia and superimposed preeclampsia) and non-obstetric renal causes (nephrotic syndrome and hemolytic uremic syndrome). Within each etiologic stratum, the associations between proteinuria severity and maternal–neonatal outcomes were evaluated using the same statistical methods as in the primary analysis, including group comparisons. These analyses were performed to determine whether the observed associations persisted within etiologically homogeneous subgroups.

Receiver operating characteristic (ROC) curve analysis was used to evaluate the predictive value of proteinuria. ROC curve analysis was used to evaluate the ability of 24 h proteinuria levels to identify pregnancies at increased risk of delivery before predefined gestational age thresholds (<32, <34, and <37 weeks), regardless of whether delivery was spontaneous or indicated. The optimal cut-off value was identified using Youden’s index.

A multivariable logistic regression analysis was performed to identify independent predictors of composite obstetric complications. In addition to nephrotic-range proteinuria (≥3 g/day), gestational age at diagnosis, estimated fetal weight (EFW) percentile, and mean arterial pressure, we also included the presence of preeclampsia (yes/no) as an adjustment variable to account for etiologic heterogeneity. Variables included in the multivariable logistic regression model were selected a priori based on clinical relevance and temporal availability at the time of initial diagnosis. Nephrotic-range proteinuria (≥3 g/day) was included as the primary exposure of interest, reflecting disease severity. Gestational age at diagnosis was incorporated as a marker of disease onset timing, given that earlier manifestation of proteinuria is consistently associated with worse outcomes in hypertensive disorders of pregnancy. Estimated fetal weight (EFW) percentile and mean arterial pressure (MAP) were included as indicators of fetal compromise and maternal hemodynamic burden, respectively, both of which are routinely available at diagnosis and clinically relevant to obstetric decision-making.

Multicollinearity among covariates was assessed using variance inflation factors (VIF), and no significant multicollinearity was detected (all VIF values < 2.5).

Internal validation of the multivariable model was performed using bootstrap resampling (1000 iterations) to estimate optimism-corrected model performance. The optimism-corrected AUC was calculated to assess the stability of discrimination.

For the multivariable logistic regression model, we reported the number of outcome events and the corresponding events-per-variable (EPV) ratio to support assessment of model stability. Only patients with complete data for all variables included in the multivariable model were analyzed (complete-case analysis). The proportion of missing data for candidate variables was low (<5%), and therefore no imputation procedures were performed. The analysis was performed as a complete-case model, and the final sample included 203 pregnancies.

Model calibration was assessed using the Hosmer–Lemeshow goodness-of-fit test and by generating a calibration plot to visually compare predicted and observed risks. The calibration plot was used to inform interpretation and is summarized narratively in the Results; the figure itself is not included in the manuscript. And the amount of explained variance was estimated with the Nagelkerke R^2^ value. A final ROC curve was created using the predicted values from the regression model.

Discriminatory performance for obstetric complications was assessed using ROC analysis, and AUC values with 95% confidence intervals were calculated for both the proteinuria-only model and the multivariable model. Since both models were generated from the same sample, the two correlated ROC curves were compared using the DeLong test to evaluate the statistical significance of the AUC difference.

No post hoc power calculation was performed. Instead, we interpreted the results primarily on the basis of effect estimates, accompanied by 95% confidence intervals where appropriate, in order to reflect the magnitude and uncertainty of the observed associations.

## 3. Results

Total of 203 pregnant women diagnosed with proteinuria between January 2020 to January 2025 were included in the study. Patients included in the study were further divided into three groups according to the severity of maternal proteinuria levels. Among participants 50 patients were diagnosed with mild proteinuria (24 h urine protein level ≥ 0.3 g/day and <3.0 g/day), 67 with severe proteinuria (≥3 gr 24 h urine protein < 5 gr) and 86 with massive proteinuria (≥5 gr/day).

Maternal demographic characteristics and perinatal outcomes of participants were presented in Table 1. The mean maternal age of patients in the mild, severe and massive proteinuria groups was 29.4 (±5.74), 30.1 (±6.46), and 28.3 (±5.16), respectively. Maternal age did not differ significantly between the three proteinuria severity groups (F_2,200_ = 1.88, *p* = 0.154). In addition, the mean BMI of the patients was 29 (±1.52), 30.6 (±5.06) and 29.9 (±3.61) in the mild, severe and massive proteinuria groups, respectively, with no statistically significant difference between them (F_2,200_ = 2.61, *p* = 0.482). Among obstetrics histories of the participants including gravidity, parity no statistically significant differences were observed between study groups (*p* = 0.260, *p* = 0.433). Mean arterial pressures of the patients were 108 (±10.4), 104 (±11.1), 106 (±11.1) in mild, severe and massive proteinuria groups, respectively. And no significant differences were observed in terms of these parameters (F_2,200_ = 1.40, *p* = 0.249). The mean gestational ages at diagnosis for the three groups were 33.7 (±1.91) weeks, 31.5 (±3.40) weeks, and 30.3 (±3.39) weeks, with a statistically significant difference (Welch’s F_2,130.5_ = 31.4, *p* = 0.001). In addition, Games–Howell post hoc comparisons revealed that the gestational age at diagnosis was lower in the severe and massive proteinuria groups compared with the mild proteinuria group, and no significant difference was observed between the severe and massive proteinuria groups (*p* = 0.001, *p* = 0.075). The mean gestational ages at delivery and birth weights in the three study groups were 37.7 (±0.65), 32.7 (±3.23) and 31.2 (±3.23) weeks 2980 (±526) gr, 1647 (±711) gr and 1343 (±613) gr, respectively, and statistically significant differences were observed with respect to these parameters between study groups (Welch’s F_2,109.7_ = 219, *p* = 0.001, F_2,200_ = 112.4, *p* = 0.001). According to Tukey’s and Games–Howell post hoc comparisons both gestational age at delivery and birth weights were lowest in the massive proteinuria group, followed by severe proteinuria group and the mild proteinuria group (*p* = 0.001, 0.010, respectively). As expected, APGAR scores differed significantly lower between the study groups (*p* = 0.001, *p* = 0.001). These above-mentioned results may be explained by the lower delivery time in massive and severe proteinuria groups compared with the mild proteinuria group. Comparison of cesarean section rates revealed no statistically significant difference among study groups (*p* = 0.631). A statistically significant difference was observed between the groups regarding the development of fetal growth restriction (FGR) (*p* = 0.001). Post hoc comparisons showed that FGR was more frequent in the severe and massive proteinuria groups compared with the mild proteinuria group (*p* = 0.001), whereas no significant difference was found between the severe and massive proteinuria groups (*p* = 0.146).

Evaluation of severity of prematurity in the study groups revealed a statistically significant difference among the study groups with respect to this parameter (*p* = 0.001). Examination of adjusted residuals performed to determine which groups differed in terms of severity of the prematurity indicated that the proportion of term delivery was significantly higher in mild proteinuria group compared with severe and massive proteinuria groups (adjusted residual = 8.4). In contrast, extremely preterm delivery rate (<28 GW) and very preterm delivery rate (≥28 GW, <32 GW) were significantly higher in massive proteinuria group compared with the mild proteinuria group (adjusted residual = 2.8, 5.2, respectively). Very preterm delivery rate was found to be higher in massive proteinuria group compared with severe proteinuria group (adjusted residual = 2.8) whereas the proportion of extremely preterm delivery was comparable between severe and massive proteinuria groups (adjusted residual = 0.7). In conclusion, the severity of prematurity tends to increase in parallel with the severity of proteinuria.

Etiological causes of proteinuria in patients and the distribution of obstetric complications were showed in Table 2. A comparison of the study groups regarding obstetric complications including placental abruption, fetal distress, and emergency cesarean section, development revealed a significant difference among the groups (*p* = 0.001). According to the post hoc-pairwise analysis, the occurrence of obstetric complications was lower in the mild proteinuria group compared with the severe and massive proteinuria groups (*p* = 0.001), while it was found to be comparable between the severe and massive proteinuria groups (*p* = 0.101). Across the proteinuria-severity groups, the distribution of the individual components of the composite obstetric outcome was as follows. Fetal distress requiring urgent delivery constituted the largest proportion of composite events in all groups, representing 6/16 (37.5%) events in the mild group, 28/46 (60.8%) in the severe group, and 53/70 (76.0%) in the massive-proteinuria group (overall 87/132; 66.0%). Placental abruption accounted for 4/16 (25.0%), 7/46 (15.2%), and 8/70 (11.4%) of events in the mild, severe, and massive groups, respectively, while HELLP syndrome occurred less frequently (≤12.5% within groups; overall 6/132; 4.5%). Severe preeclampsia contributed 25.0%, 13.1%, and 7.0% of composite events across the three groups, and intrauterine fetal death was uncommon (≤6.5% in any group). These figures indicate that the majority of composite events were related to fetal-distress-based delivery indications, whereas the more objective complications (placental abruption, HELLP syndrome, and IUFD) represented a smaller proportion of the total events. Similarly, the rate of requirement for antihypertensive therapy was higher in the severe and massive proteinuria groups compared with the mild proteinuria group (*p* = 0.017), whereas no difference was observed between the severe and massive proteinuria groups (*p* = 0.866). The comparison of the etiological causes of patients according to the severity of proteinuria demonstrated a statistically significant difference between the groups (*p* = 0.001). Based on the adjusted residuals analysis performed to determine which etiological causes differed between the groups, the rate of nephrotic syndrome was higher in the massive proteinuria group compared with the mild group (adjusted residual = 2.5), whereas the rate of gestational proteinuria was higher in the mild proteinuria group (adjusted residual = 4.1). Similarly, when the severe and massive proteinuria groups were compared, nephrotic syndrome was a more frequent etiological cause in the massive proteinuria group (adjusted residual = 2.5). When the entire patient population was considered, the rates of preeclampsia and superimposed preeclampsia were identified as 56%, 76%, and 72% in the mild, severe, and massive proteinuria groups, respectively, with obstetric causes being the most common etiological factors overall. However, non-obstetric causes, such as HUS and nephrotic syndrome, were more frequently identified in the severe and massive proteinuria groups. Overall, among all patients, the most common obstetric complication was fetal distress (65%), followed by placental abruption (14.4%) and severe preeclampsia (11.4%), respectively. The intergroup comparison of the types of obstetric complications also revealed a statistically significant difference (*p* = 0.001). Based on the evaluation of the adjusted residuals performed to determine which obstetric complications differed between the groups, the rate of absence of obstetric complications was higher in the mild proteinuria group compared with the massive proteinuria group (adjusted residual = 5.7), while the rate of fetal distress was higher in the massive proteinuria group (adjusted residual = 5.8). In contrast, no difference was observed between the severe and massive proteinuria groups in terms of obstetric complications (*p* = 0.363).

To assess the robustness of the observed dose–response relationship, sensitivity analyses were performed and presented in Table 3. First, analyses were restricted to patients with obstetric causes of proteinuria, including preeclampsia and superimposed preeclampsia, excluding cases with nephrotic syndrome and hemolytic uremic syndrome. In this restricted cohort, increasing proteinuria severity remained significantly associated with higher rates of obstetric complications, earlier gestational age at delivery, and lower birth weight (all *p* < 0.05).

Second, when analyses were limited to patients diagnosed with preeclampsia only, the dose-dependent relationship between proteinuria severity and adverse maternal–neonatal outcomes persisted, with nephrotic-range proteinuria remaining an independent predictor of composite obstetric complications.

To further explore whether the observed associations were driven by underlying diagnosis rather than proteinuria severity alone, stratified analyses were performed according to major etiologic categories and were presented in Table 4. Patients were grouped as having hypertensive disorders of pregnancy (preeclampsia and superimposed preeclampsia) or non-obstetric renal causes (nephrotic syndrome and hemolytic uremic syndrome).

Within the hypertensive disorders group, increasing proteinuria severity remained significantly associated with higher rates of obstetric complications, earlier gestational age at delivery, lower birth weight, and increased frequency of fetal growth restriction (all *p* < 0.05).

In contrast, within the non-obstetric renal subgroup, proteinuria severity was not significantly associated with the obstetric complications (*p* = 0.193). However, higher proteinuria severity was associated with earlier gestational age at delivery and lower birth weight (*p* < 0.005), indicating that proteinuria retains prognostic relevance for delivery timing and neonatal growth-related outcomes even in renal causes of proteinuria.

Correlation analysis (Table 5) revealed that the 24 h urine protein level had a statistically significant, moderate negative correlation with birth weight (r = −0.44, *p* < 0.001) and gestational age at delivery (r = −0.42, *p* < 0.001). It also showed significant negative correlations with 1 and 5 min APGAR scores, EFW percentile, and gestational age at diagnosis. No significant correlation was found with mean arterial pressure (*p* = 0.199).

**Figure 2:**
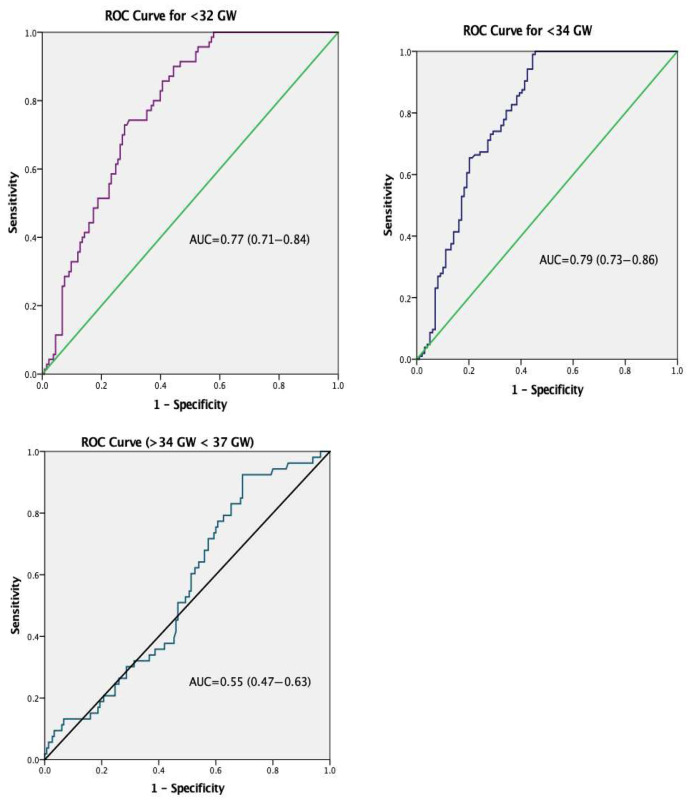
The receiver operating characteristic (ROC) curve analysis of maternal 24 h urine protein levels for identification of increased risk of early delivery occuring before (<32 GW), (<34 GW) and late delivery (≥34 GW < 37 GW) in patients diagnosed with proteinuria. AUC: area under the curve; GW: gestational week; Very preterm: delivery ≥ 28 GW < 32 GW; Moderate preterm: delivery ≥ 32 GW < 34 GW; Late preterm: delivery ≥ 34 GW < 37 GW; Term: ≥37 GW.

Figure 2 presents the ROC curves evaluating the risk-stratification performance of the amount of protein in the 24 h urine collection for identification of increased risk of early delivery occurring before 32 gestational weeks, between 32–34 gestational weeks, and between 34–37 gestational weeks. Because preterm delivery in hypertensive disorders of pregnancy is frequently iatrogenic, the outcome of interest was defined as delivery before specific gestational age thresholds, rather than spontaneous preterm birth. Thus, ROC analyses should be interpreted as reflecting the ability of proteinuria severity to stratify the risk of early delivery—whether spontaneous or clinically indicated—rather than causal prediction of spontaneous preterm labor. All ROC analyses were based on the initial 24 h proteinuria measurement obtained at diagnosis, prior to delivery, to minimize potential bias related to disease progression or delivery decisions. Based on the ROC curve analyses, the amount of proteinuria in the 24 h urine collection was shown to identify increased risk of early delivery occurring before 32 gestational weeks (AUC: 0.77; 95% CI: 0.71–0.84) and 34 gestational weeks (AUC: 0.79; 95% CI: 0.73–0.86). For deliveries occurring before 34 weeks of gestation, the ROC analysis identified a proteinuria threshold of ≥3.1 g/day (Youden’s index = 0.54), which demonstrated a sensitivity of 97% and a specificity of 56% for discriminating pregnancies at increased risk of earlier delivery, rather than predicting preterm birth in a causal sense.

The results of multivariate logistic regression analyses incorporating presence of nephrotic-range proteinuria (≥3 g/day), GA at diagnosis (weeks), EFW percentile, mean arterial pressure and presence of preeclampsia to identify factors associated with obstetric complications were presented in Table 6.

The multivariable model included 132 composite obstetric-complication events, corresponding to an EPV of approximately 26.4 per covariate (5 predictors: nephrotic-range proteinuria ≥ 3 g/day, gestational age at diagnosis, estimated fetal-weight percentile, mean arterial pressure, and presence of preeclampsia), and 203 women were included in the complete-case analysis. Model calibration was acceptable according to the Hosmer–Lemeshow goodness-of-fit test (*p* = 0.85), and inspection of the calibration plot showed overall good agreement between predicted and observed event probabilities across risk strata, with only minor deviation in the higher-risk range that did not materially affect model performance (Supplementary figure available on request). In addition, we achieved a moderate Nagelkerke level (Nagelkerke R^2^ = 0.32), which explains 32% of the variance in the dependent variable. In multivariate logistic regression analyses, the GA at diagnosis (OR = 0.76, 95% CI: 0.66–0.87, *p* = 0.001), and presence of nephrotic-range proteinuria (≥3 g/day) (OR = 3.12, 95% CI: 1.41–6.92, *p* = 0.005) have an effect on occurrence of obstetric complications. On the other hand, EFW percentile levels, mean arterial pressures and presence of preeclampsia (including superimposed preeclampsia) had no significant effect on the presence of obstetric complications rate (*p* = 0.119, *p* = 0.832, *p* = 0.440, respectively). To account for etiologic heterogeneity, the multivariable model was additionally adjusted for the presence of preeclampsia (including superimposed preeclampsia). After adjustment, nephrotic-range proteinuria remained independently associated with composite obstetric complications, indicating that proteinuria severity contributes prognostic information beyond the diagnosis of preeclampsia alone.

**Figure 3:**
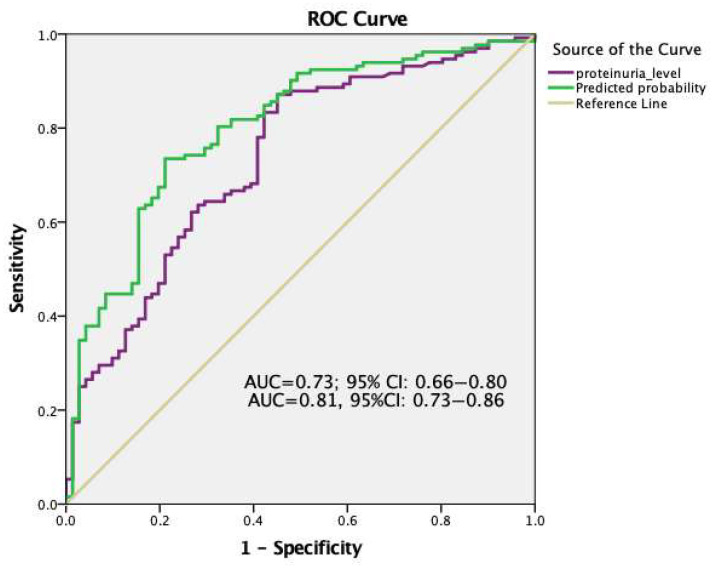
The receiver operating characteristic (ROC) curve analyses of maternal 24 h urine protein levels and the combined model derived from the logistic regression analysis for prediction of obstetric complications in patients diagnosed with proteinuria. AUC: area under the curve.

As a result of our ROC analysis of 24 h urine protein levels demonstrated moderate discrimination for obstetric complications (AUC = 0.73; 95% CI: 0.66–0.80) (Figure 3). Optimal cut-off for proteinuria levels corresponding to highest Youden’s index (0.42) was determined ≥3.1 g/day with sensitivity of 87% and with a specificity 55%. Additionally, the ROC analysis derived from this combined model is presented in Figure 3. According to this ROC analysis, a high AUC value was obtained, and the multivariable model demonstrated improved discriminatory ability compared with proteinuria alone. (AUC = 0.81, 95% CI: 0.73–0.86). After bootstrap internal validation, the optimism-corrected AUC of the multivariable model remained robust at 0.79, indicating minimal overfitting.

Moreover, ROC curves for the combined model and for 24 h proteinuria alone are presented in Figure 3. In ROC analysis, the model including continuous 24 h proteinuria alone yielded an AUC of 0.73 (95% CI, 0.66–0.80) for obstetric complications. The multivariable model incorporating nephrotic-range proteinuria, mean arterial pressure, gestational age at diagnosis, EFW percentile, and preeclampsia status demonstrated an AUC of 0.81 (95% CI, 0.73–0.86). Because both models were developed from the same dataset, their AUCs were compared using the DeLong test. Although the combined model showed a numerically higher AUC, the difference between the two ROC curves did not reach statistical significance (DeLong z = 0.82, *p* = 0.41).

Based on the ROC analyses, proteinuria levels exceeding the nephrotic range (≥3 g/day) may be useful for identifying pregnancies at higher risk of obstetric complications and earlier delivery.

## 4. Discussion

This study provides a detailed analysis of the relationship between maternal proteinuria severity and various obstetric and neonatal outcomes. Our main findings show a clear, dose-dependent association between increasing proteinuria levels and a higher frequency of adverse outcomes. In particular, patients with severe (≥3 g/day) and massive (≥5 g/day) proteinuria had significantly higher rates of obstetric complications, delivered at earlier gestational ages, had lower birth weights, and required antihypertensive treatment more often compared with patients with mild proteinuria. In addition, our analysis demonstrated that a multivariable model including both nephrotic-range proteinuria and basic clinical parameters had better risk-stratification performance than using proteinuria levels alone.

Notably, the independent association of nephrotic-range proteinuria with obstetric complications persisted even after adjustment for preeclampsia status, supporting proteinuria severity as a prognostic marker beyond etiologic classification. Given the etiologic heterogeneity of proteinuria in pregnancy, we further explored whether this association was robust across diagnostic subgroups using sensitivity and stratified analyses. Sensitivity analyses restricted to obstetric causes of proteinuria confirmed the robustness of the primary findings. Stratified analyses further demonstrated outcome-specific heterogeneity: while proteinuria severity remained associated with adverse outcomes within hypertensive disorders of pregnancy, in non-obstetric renal causes it was associated with earlier delivery and lower birth weight but not with the composite obstetric complication outcome. These findings suggest that proteinuria severity may convey prognostic information beyond diagnostic category, particularly for delivery timing and fetal growth–related outcomes.

We acknowledge that in hypertensive disorders of pregnancy, preterm delivery is frequently iatrogenic and driven by clinical decision-making rather than spontaneous disease progression. Consequently, the association between proteinuria severity and preterm birth may be influenced by reverse causality, whereby more severe disease prompts earlier delivery. To mitigate this concern, proteinuria measurements used in our analyses were obtained at the time of diagnosis and before delivery decisions were made. Nevertheless, our findings should be interpreted as reflecting risk stratification rather than true causal prediction of spontaneous preterm birth. In addition, A time-to-event approach, such as survival analysis, could theoretically provide a more nuanced assessment of the relationship between proteinuria severity and time to delivery. However, given the retrospective design and the strong influence of clinician-driven delivery decisions, such analyses may also be subject to similar limitations. This should be considered when interpreting the risk-stratification performance of proteinuria in this context.

One of the main findings of our study is that severity of proteinuria has a valuable prognostic importance for adverse pregnancy outcomes. This finding is consistent with and extends the results of several previous studies [12,13,14]. We observed a clear risk gradient, with higher levels of protein excretion being more strongly associated with fetal growth restriction, preterm birth, and lower APGAR scores. This dose–response relationship is in line with the study by Hu et al. (2023), which reported that moderate and severe proteinuria were strongly linked to poor perinatal outcomes [12]. Similarly, Tanacan et al. (2019) found that complications such as HELLP syndrome and placental abruption were more common in patients with severe and massive proteinuria [18]. By supporting these findings, our study shows that as proteinuria increases from mild to massive, the risks for both maternal and fetal complications rise significantly.

Interestingly, although we found clear differences in outcomes between the mild and the severe/massive proteinuria groups, the differences between the severe and massive proteinuria groups were not statistically significant in terms of obstetric complication rates and the need for antihypertensive treatment. This finding may suggest the presence of a possible clinical threshold at the nephrotic range (≥3 g/day), beyond which the risk of adverse outcomes increases markedly. Once this threshold is reached, further increases in proteinuria may add limited additional prognostic information. This is clinically important, as it indicates that crossing into the nephrotic range may be a key point for risk stratification in clinical practice.

Our findings also emphasize the heterogeneous etiology of proteinuria in pregnancy. While preeclampsia and preeclampsia superimposed on chronic hypertension remained the most frequent causes in all severity groups, their distribution varied according to proteinuria level. Gestational proteinuria was predominantly observed in the mild proteinuria group, which is consistent with its typically favorable clinical course [19]. In contrast, non-obstetric conditions—most notably nephrotic syndrome—were considerably more common among patients with massive proteinuria. This distinction is clinically relevant, as it indicates that severe proteinuria should not automatically be attributed to preeclampsia alone, but rather should trigger evaluation for alternative diagnoses, including primary renal disease [9]. Given that the clinical management and long-term outcomes of nephrotic syndrome differ markedly from those of preeclampsia, our results highlight the importance of thorough etiological assessment in pregnancies complicated by massive proteinuria [20].

In our study population, the most common obstetric complication was fetal distress, and its prevalence increased significantly with greater proteinuria severity. This finding may be related to underlying placental pathology seen in severe cases of proteinuria. Genest et al. (2021) reported that more severe proteinuria in preeclampsia is associated with a higher prevalence of maternal vascular malperfusion in the placenta [21]. Endothelial dysfunction and systemic inflammation, which contribute to both glomerular injury and proteinuria, are also likely to impair placental function. This impaired placental function may lead to chronic fetal stress, presenting as fetal growth restriction (FGR) and, in acute situations, fetal distress.

Previous studies in the literature have also shown that the rate of adverse pregnancy outcomes increases as the severity of proteinuria increases. In line with our findings, a study conducted by Yılmaz Baran et al. in 2020 reported that higher levels of proteinuria were associated with worse pregnancy outcomes [22]. Similarly, the study by Piccoli et al. observed increased rates of preterm birth and neonatal intensive care unit admission, consistent with the results of our study [23]. Bramham et al. (2013) reported that pregnant women with proteinuria greater than 0.5 g/day had worse pregnancy outcomes than those with proteinuria of 0.3 g/day, including higher rates of preterm delivery, severe hypertension, and the need for magnesium sulphate treatment during pregnancy, suggesting that proteinuria may demonstrate discriminatory ability for adverse-outcome risk [24].

Although the clinical value of proteinuria measurement in the management of preeclampsia remains debated [1,11], our findings offer a different perspective. We found that, 24 h proteinuria levels showed moderate discrimination in identifying pregnancies at higher risk of adverse outcomes. Our ROC analysis identified an optimal cut-off value of approximately 3.1 g/day for identifying pregnancies at higher risk of adverse outcomes, which closely aligns with the commonly accepted definition of nephrotic-range proteinuria. The ROC-derived cut-off value was close to the predefined nephrotic-range threshold, and should therefore be interpreted in the context of existing clinical classifications rather than as a novel severity definition. This may suggest that this threshold is not merely a diagnostic convention, but also represents a clinically meaningful increase in risk. Moreover, these findings should be interpreted as risk stratification based on observational associations, not as causal prediction.

In our study, the multivariable model that included nephrotic-range proteinuria (≥3 g/day) and gestational age at diagnosis showed a numerically higher AUC (AUC 0.73 vs. 0.81) than the proteinuria-only model; however, this difference was not statistically significant. Rather than indicating statistical superiority, this finding may simply reflect the additional clinical information captured when multiple parameters are considered together. Accordingly, the ROC results should be interpreted as supportive and exploratory, complementing the primary analyses rather than serving as their main basis. In addition, gestational age at diagnosis was independently associated with adverse outcomes alongside proteinuria, suggesting that earlier disease presentation may be linked to a higher-risk clinical profile, a pattern consistent with previous reports [8].

We acknowledge that proteinuria in pregnancy arises from heterogeneous etiological pathways, including placental disease, chronic or acute renal pathology, and benign gestational conditions. As demonstrated in our cohort, etiological distribution varied significantly across proteinuria severity groups, with gestational proteinuria clustering in the mild group and nephrotic syndrome being more prevalent among patients with massive proteinuria. This raises the possibility that adverse outcomes may be partially driven by underlying diagnosis rather than protein quantity alone.

However, our objective was not to imply a uniform pathophysiological mechanism across these conditions, but rather to evaluate whether the degree of proteinuria—regardless of etiology—serves as a clinically pragmatic marker of risk in a real-world tertiary care population. Importantly, even after accounting for etiological heterogeneity, nephrotic-range proteinuria remained strongly associated with adverse outcomes, supporting its role as a meaningful indicator of disease severity and pregnancy risk.

This study has several strengths, including a relatively large cohort for a single-center study, which provides a good level of consistency in diagnostic criteria and clinical management protocols. The detailed stratification of proteinuria severity allowed for a thorough assessment of its impact on outcomes. In addition, the use of robust statistical methods, such as multivariable logistic regression further strengthens the validity of our risk-stratification model.

Despite its strengths, this study has several limitations. Retrospective design of the study is the one of the limitations. In addition, only women with proteinuria were included, and there was no control group with normal urinary protein levels. Another important limitation of this study is its single-center, tertiary referral design. As a tertiary perinatology clinic, our institution is more likely to receive referrals of pregnancies complicated by severe proteinuria or associated maternal–fetal conditions, whereas women with mild proteinuria and an uncomplicated clinical course may deliver at secondary or primary care centers. This referral pattern may have enriched our cohort for more severe cases, potentially inflating absolute event rates and amplifying the observed dose–response relationship between proteinuria severity and adverse outcomes. However, all eligible women with documented proteinuria who delivered at our center during the study period were consecutively included, and the direction and magnitude of our findings are consistent with previously published studies from diverse clinical settings. Nevertheless, caution is warranted when generalizing our results to broader obstetric populations. Because some components of the composite obstetric outcome (particularly urgent delivery for fetal distress) may be influenced by clinician decision thresholds, the association between proteinuria severity and this outcome should be interpreted within a risk-stratification rather than causal framework, and may partly reflect management-driven intervention rather than purely biological progression. A further limitation of our study is that we did not perform a post hoc power analysis. Such analyses are generally considered to have limited interpretive value in retrospective observational designs and may be misleading when interpreted alongside effect estimates. For this reason, we based the interpretation of our results on effect sizes and their 95% confidence intervals, which more appropriately reflect the magnitude and precision of the observed associations.

## 5. Conclusions

In conclusion, our findings may suggest that increasing proteinuria severity is associated with a graded increase in adverse maternal and neonatal outcomes, and that reaching nephrotic-range levels (≥3 g/day) may represent a potentially meaningful clinical threshold in this population. While 24 h proteinuria remains a useful prognostic marker, its discriminatory performance appeared numerically higher when considered together with other clinically relevant parameters, particularly gestational age at diagnosis; however, this difference was not statistically significant and therefore represents a modest, incremental improvement in discrimination rather than a major enhancement in predictive performance. These observations may support a more nuanced, multifactorial approach to risk stratification and follow-up in pregnant women with proteinuria, rather than reliance on a single parameter alone. Future prospective, multicenter studies are needed to confirm these associations and to further evaluate the clinical applicability of such integrated risk-assessment approaches across diverse patient populations.

## Figures and Tables

**Table 1:** The demographic characteristics and the perinatal outcomes of the pregnant women diagnosed with proteinuria.

		Proteinuria 24 h Urine (mg/dL)				
		Mild (≥0.3 gr 24 h Proteinuria < 3 gr) (*n* = 50)	Severe (≥3 gr 24 h Proteinuria < 5 gr) (*n* = 67)	Massive (≥5 gr 24 h Proteinuria) (*n* = 86)	Total (*n* = 203)	*p* Values ^a^
**Age (years) ^b^**		29.4 (±5.74)	30.1 (±6.46)	28.3 (±5.16)	29.2 (±5.79)	0.154
**Gravida (*n*) ^c^**		2 (1–3)	1 (1–5)	2 (1–8)	2 (1–8)	0.260
**Parity (*n*) ^c^**		1 (0–3)	0 (0–4)	0 (0–6)	0 (0–6)	0.433
**BMI (kg/m^2^) ^b^**		29 (±1.52)	30.6(±5.06)	29.9 (±3.61)	29.9 (±3.85)	0.482
**Mean arterial pressure ^b^**		108 (±10.04)	104 (±11.06)	106 (±11.1)	106 (±10.8)	0.249
**GA at diagnosis (weeks) ^b^**		33.7 (±1.91)	31.5 (±3.40)	30.3 (±3.39)	31.5 (±3.38)	**0.001**
**GA at delivery (weeks) (weeks) ^b^**		37.7 (±0.65)	32.7 (±3.23)	31.2 (±3.23)	33.3 (±3.81)	**0.001**
**Birth weight (g) ^b^**		2980 (±526)	1647 (±711)	1343 (±613)	1847 (±911)	**0.001**
**APGAR 1. minute ^c^**		8 (7–9)	7 (0–9)	6 (0–9)	6 (0–9)	**0.001**
**APGAR 5. minute ^c^**		9 (8–10)	8 (0–10)	8 (0–10)	8 (0–10)	**0.001**
**Presence of FGR ^d^**		17 (34%)	45 (67.1%)	67 (77.9%)	129 (63.5%)	**0.001**
**Birth method ^d^**	**SVB**	6 (12%)	6 (9%)	5 (6%)	17 (9%)	0.631
	**CS**	44 (88%)	61 (91%)	81 (94)	186 (91%)	
**Severity of prematurity ^d^**						**0.001**
	**Extremely preterm**	0 (0%)	7 (10.45%)	12 (13.9%)	19 (9.36%)	
	**Very preterm**	0 (0%)	13 (19.4%)	35 (40.7%)	48 (23.6%)	
	**Moderate preterm**	0 (0%)	20 (29.8%)	21 (24.4%)	41 (20.2%)	
	**Late preterm**	4 (8%)	18 (26.8%)	15 (17.4%)	37 (18.3%)	
	**Term**	46 (92%)	9 (13.4%)	3 (3.50%)	58 (28.6%)	

BMI: body mass index; GA: gestational age; SVB: spontaneous vaginal birth; CS: cesarean section; FGR: fetal growth restriction; Extremely preterm: delivery < 28. Gestational week; Very preterm: delivery ≥ 28 GW < 32 GW; Moderate preterm: delivery ≥ 32 gw < 34 GW; Late preterm: delivery ≥ 34 GW < 37 GW; Term: ≥37 GW. ^a^ Level of significance *p* < 0.05. ^b^ Continuous variables that were normally distributed are expressed as mean ± standard deviation and were compared using One-way ANOVA test with Tukey’s and Games–Howell post hoc tests for pairwise comparison. ^c^ Data that were not normally distributed are expressed as median (minimum-maximum) and were compared using Kruskal–Wallis H test with post hoc Mann–Whitney U test. ^d^ Categorical variables were presented as percentage (%) and count (*n*) and were compared using the Chi-Square test and for pairwise comparison Pairwise Chi-Square test and adjusted standardized residuals were used. Bold values indicate statistically significant results (*p* < 0.05).

**Table 2:** Etiological causes of proteinuria in patients and the distribution of obstetric complications.

		Proteinuria 24 h Urine (mg/dL)				
		Mild (≥0.3 gr 24 h Proteinuria < 3 gr) (*n* = 50)	Severe (≥3 gr 24 h Proteinuria < 5 gr) (*n* = 67)	Massive (≥5 gr 24 h Proteinuria) (*n* = 86)	Total (*n* = 203)	*p* Values ^a^
**Presence of obstetrics** **complication ^b^**	**Presence**	16 (32%)	46 (69%)	70 (81%)	132 (65%)	**0.001**
	**Absence**	34 (68%)	21 (31%)	16 (19%)	71 (35%)	
**Requirement for AHT ^b^**	**Yes**	24 (48%)	45 (67%)	60 (70%)	129 (64%)	**0.017**
	**No**	26 (52%)	22 (33%)	26 (30%)	74 (36%)	
**Etiology ^b^**	**Preeclampsia**	28 (56%)	51 (76%)	53 (62%)	132 (65%)	**0.001**
	**Superimposed preeclampsia**	8 (16%)	2 (3%)	11 (13%)	21 (11%)	
	**Gestational proteinuria**	11 (22%)	6 (9%)	1 (1%)	18 (9%)	
	**Nephrotic syndrome**	3 (6%)	5 (7.5%)	19 (22%)	27 (13%)	
	**HUS**	0	3 (4.5%)	2 (2.3%)	5 (2.5%)	
**Type of obstetric complications ^b^**						**0.001**
	**Decolman placenta**	4 (25%)	7 (15.2%)	8 (11.4%)	19 (14%)	
	**Fetal distress**	6 (37.5%)	28 (60.8%)	53 (76%)	87 (66%)	
	**HELLP**	2 (12.5%)	2 (4.3%)	2 (2%)	6 (4.5%)	
	**Severe preeclampsia**	4 (25%)	6 (13.1%)	5 (7%)	15 (11.4%)	
	**Intrauterine fetal death**	0 (0%)	3 (6.5%)	2 (3%)	5 (3.8%)	

HUS: hemolytic uremic syndrome; AHT: anti-hypertensive treatment; Extremely preterm: delivery < 28. Gestational week; Very preterm: delivery ≥ 28 GW < 32 GW; Moderate preterm: delivery ≥ 32 gw < 34 GW; Late preterm: delivery ≥ 34 GW < 37 GW; Term: ≥ 37 GW; HELLP: hemolysis, elevated liver enzymes, lower platelet. ^a^ Level of significance *p* < 0.05. ^b^ Categorical variables were presented as percentage (%) and count (*n*) and were compared using the Chi-Square test and for pairwise comparison Pairwise Chi-Square test and adjusted standardized residuals were used. Bold values indicate statistically significant results (*p* < 0.05).

**Table 3:** Sensitivity analyses of maternal and neonatal outcomes according to proteinuria severity after exclusion of non-obstetric causes. GA: gestational age.

		Proteinuria 24 h Urine (mg/dL)				
		Mild (≥0.3 gr 24 h Proteinuria < 3 gr) (*n* = 47)	Severe (≥3 gr 24 h Proteinuria < 5 gr) (*n* = 59)	Massive (≥5 gr 24 h Proteinuria) (*n* = 65)	Total (*n* = 171)	*p* Values ^a^
**Presence of obstetrics** **complication ^c^**	**Presence**	15 (32%)	42 (71%)	54 (84%)	111 (65%)	**0.001**
	**Absence**	32 (68%)	17 (29%)	11 (16%)	60 (35%)	
**Birth weight (g) ^b^**		2986 (±522)	1622 (±712)	1330 (±519)	1886 (±908)	**0.001**
**GA at delivery (weeks) ^b^**		37.7 (±0.66)	32.6 (±3.24)	31.4 (±2.79)	33.5 (±3.24)	**0.001**

^a^ Level of significance *p* < 0.05. ^b^ Continuous variables that were normally distributed are expressed as mean ± standard deviation and were compared using One-way ANOVA test with Tukey’s and Games–Howell post hoc tests for pairwise comparison. ^c^ Categorical variables were presented as percentage (%) and count (*n*) and were compared using the Chi-Square test and for pairwise comparison Pairwise Chi-Square test and adjusted standardized residuals were used. Bold values indicate statistically significant results (*p* < 0.05).

**Table 4:** Stratified analyses of maternal and neonatal outcomes according to proteinuria severity in non-obstetric causes of proteinuria. GA: gestational age.

		Proteinuria 24 h Urine (mg/dL)				
		Mild (≥0.3 gr 24 h Proteinuria < 3 gr) (*n* = 3)	Severe (≥3 gr 24 h Proteinuria < 5 gr) (*n* = 8)	Massive (≥5 gr 24 h Proteinuria) (*n* = 21)	Total (*n* = 32)	*p* Values ^a^
**Presence of obstetrics** **complication ^b^**	**Presence**	1 (34%)	4 (50%)	16 (76%)	21 (65%)	0.193
	**Absence**	2 (66%)	4 (50%)	5 (24%)	11 (35%)	
**Birth weight (g) ^c^**		2670 (2350–3720)	1940 (700–3090)	1340 (250–3175)	1740 (250–3720)	**0.038**
**GA at delivery (weeks) ^c^**		38 (37–38.1)	34.3 (27.6–37)	30.5 (23.1–37)	32.3 (23.1–38.1)	**0.007**

^a^ Level of significance *p* < 0.05. ^b^ Categorical variables were presented as percentage (%) and count (*n*) and were compared using the Chi-Square test and for pairwise comparison Pairwise Chi-Square test and adjusted standardized residuals were used. ^c^ Data that were not normally distributed are expressed as median (minimum-maximum) and were compared using Kruskal–Wallis H test with post hoc Mann–Whitney U test. Bold values indicate statistically significant results (*p* < 0.05).

**Table 5:** Correlation analyses between 24 h urine protein levels and clinical parameters in the study population.

Proteinuria 24 h Urine (g/Day)		
	r	*p* ^a^
**EFW (percentile) ^c^**	−0.36	**0.001**
**GA at delivery (weeks) ^b^**	−0.42	**0.001**
**GA at diagnosis (weeks) ^b^**	−0.25	**0.001**
**Mean arterial pressure ^b^**	−0.09	0.199
**Birth weight (gr) ^b^**	−0.44	**0.001**
**APGAR 1st minute ^c^**	−0.43	**0.001**
**APGAR 5th minute ^c^**	−0.34	**0.001**

GA: gestational age. ^a^ Level of significance *p* < 0.05. ^b^ r = Pearson’s correlation. ^c^ r = Spearman’s correlation. Bold values indicate statistically significant results (*p* < 0.05).

**Table 6:** Multivariate Logistic regression analysis of factors affecting occurrence of obstetric complications in the study population.

Parameters			Multivariate Analysis			
	β (Coefficient)	Standard Error	Wald Value	*p*-Values ^a^	OR	95% Confidence Interval
**Presence of nephrotic-range proteinuria (≥3 g/day) ^b^**	1.14	0.40	7.88	**0.005**	3.12	1.41–6.92
**GA at diagnosis (weeks)**	−0.27	0.06	15.8	**0.001**	0.76	0.66–0.87
**EFW Percentile**	−0.01	0.01	2.43	0.119	0.98	0.96–1.04
**Mean arterial pressure**	−0.01	0.01	0.04	0.832	0.99	0.96–1.02
**Presence of preeclampsia ^b^**	0.30	0.39	0.59	0.440	1.35	0.62–2.93

GA: gestational age; EFW: estimated fetal weight. *p* value, odds ratio, and 95% confidence interval of the odds ratio were calculated using multivariate logistic regression analysis, comparing data among the study population. ^a^ Level of significance *p* < 0.05. ^b^ OR value was specified to reference group (for categorical covariates). Bold values indicate statistically significant results (*p* < 0.05).

## Data Availability

Dataset available on request from the authors.
